# Management of Patients with *Candida auris* Fungemia at Community Hospital, Brooklyn, New York, USA, 2016–2018^1^

**DOI:** 10.3201/eid2503.180927

**Published:** 2019-03

**Authors:** Jenny YeiSol Park, Nicole Bradley, Steven Brooks, Sibte Burney, Chanie Wassner

**Affiliations:** Kingsbrook Jewish Medical Center, Brooklyn, New York, USA

**Keywords:** fungi, fungemia, antifungal drugs, antifungal resistance, antifungal susceptibility, antimicrobial resistance, Candida auris, infection, management of patients, mortality, risk factors, community hospital, Brooklyn, New York, United States

## Abstract

*Candida auris* is an emerging fungus that can cause invasive infections. It is associated with high mortality rates and resistance to multiple classes of antifungal drugs and is difficult to identify with standard laboratory methods. We describe the management and outcomes of 9 patients with *C*. *auris* fungemia in Brooklyn, New York, USA.

*Candida auris* is an emerging fungus that can cause invasive infections associated with high mortality rates and is often resistant to multiple classes of antifungal drugs. Risk factors for infection include nursing home exposure; invasive devices, such as tracheostomy tubes or percutaneous endoscopic gastrostomy tubes; immunocompromised status; and use of broad-spectrum antimicrobial drugs (*1*). On the basis of limited data available, echinocandins are recommended as initial therapy for *C. auris* infection (*2*). We review the management of 9 case-patients who had *C. auris* fungemia at a 300-bed community hospital, attached to a 450-bed nursing home, in Brooklyn, NY, USA. There have been 9 occurrences of C. *auris* fungemia at this institution since 2016.

Our case series demonstrates the complex patient population at risk for invasive infection with *C. auris*. Patients infected were generally >70 years of age and had multiple chronic concurrent conditions (Appendix Table). Most patients came from nursing homes, and more than half had invasive devices, such as tracheostomies or percutaneous endoscopic gastrostomy tubes, placing them at high risk for infection at baseline.

In addition, each patient had a recent history of broad-spectrum antimicrobial drug use; many had concomitant resistant organisms isolated and received concomitant antimicrobial drug therapy during their *C. auris* treatment course. The most common antimicrobial drugs used were meropenem, polymyxin B, and vancomycin. Common bacteria isolated were *Acinetobacter* sp., *Pseudomonas aeruginosa,* and *Klebsiella pneumoniae.* Four patients were admitted to the medical intensive care unit; 2 had prolonged stays in the medical intensive care unit before development of candidemia.

The time from hospitalization to initial infection with *C. auris* varied among the patients. Approximately 60% of the patients came to the hospital with positive blood cultures on day 1, and fungemia developed in the remaining patients after prolonged hospitalization. Most patients had documented clearance of their blood cultures within 3–5 days of initial isolation. However, 1 patient had a second episode of *C. auris* fungemia several weeks after he was initially given treatment and documented to have clearance of blood cultures.

All patients were given micafungin as first-line therapy for an average duration of 22 days. The most common dose used was 100 mg/day of intravenous micafungin. Two of the 9 patients required liposomal amphotericin B after failing to respond to micafungin therapy. Both of these patients remained persistently febrile while receiving micafungin monotherapy; 1 was the patient with 2 episodes of *C. auris* fungemia. The average duration of amphotericin B was 19 days. The in-hospital mortality rate was 22%. Of the 7 patients who were discharged, 43% were discharged to a palliative care service. The average duration of hospitalization for these patients was 65 days.

Limited information is available on interpretation of MIC data for *C. auris* because the Clinical Laboratory Standards Institute (https://clsi.org) does not have breakpoints specific for *C. auris*. Antifungal susceptibility data were determined for each of the patients included in this case series (Appendix Figure). All antifungal susceptibility tests were performed by the Wadsworth Laboratory, the New York State Department of Health Reference Laboratory (https://www.wadsworth.org). Most susceptibility information was not available throughout the course of treatment. Given difficult identification of the organism by using standard laboratory techniques, timely identification and antimicrobial susceptibility information continues to be a challenge when managing patients with invasive *C. auris* (*3*).

All isolates were markedly resistant to fluconazole, and ≈40% were resistant to liposomal amphotericin B (Appendix Figure). This second finding is of particular concern because liposomal amphotericin B is the recommended second-line agent for management of *C. auris* in the setting of micafungin failure. On the basis of the resistance patterns at this hospital, patients who failed monotherapy with micafungin had lisposomal amphoterin B added rather than switching therapy completely.

We encourage clinicians treating *C. auris* infections to consider combination therapy with micafungin plus liposomal amphotericin B in patients who fail monotherapy with micafungin. Laboratory limitations mean that timely identification and susceptibility testing of *C. auris* might not always be possible, and clinicians might often have to defer to local or national epidemiology trends to make the most up-to-date decisions. It is essential to notify the department of health of new cases of infection with *C. auris* as soon as possible and to educate healthcare personnel to help minimize spread. Clinicians should focus on identifying and minimizing risk factors for acquisition of *C. auris* and prevention of spread through enhanced infection control procedures.

Appendix Additional information on management of patients with *Candida auris* fungemia at community hospital, Brooklyn, New York, USA, 2016–2018.
