# Supplementary material for: Management of Patients with Candida auris Fungemia at Community Hospital, Brooklyn, New York, USA, 2016–2018
Source: Emerg Infect Dis. 2019 Mar;25(3):601–2. doi: 10.3201/eid2503.180927 (PMC6390749; doi:10.3201/eid2503.180927)
Supplement: Appendix — Additional information on management of patients with Candida auris fungemia at community hospital, Brooklyn, New York, USA, 2016–2018. [file 18-0927-Techapp-s1.pdf]

Article DOI: https://doi.org/10.3201/eid2503.180927

Management of Patients with *Candida auris* Fungemia at Community Hospital, Brooklyn, New York, USA, 2016–2018

Appendix

Appendix Table. Characteristics for 9 case-patients during management of *Candida auris* fungemia at community hospital, Brooklyn, New York, USA, 2016–2018*

Case-patient	Age, y/sex	Medical history	Admitted from nursing home	Presence of invasive device	Recent broad-spectrum antimicrobial drug
Baseline demographics					
1	80/F	Hypertension, diabetes, atrial fibrillation, multiple myeloma, respiratory failure	Yes	No	Yes
2	73/M	Hyperlipidemia, diabetes, congestive heart failure, chronic kidney disease, atrial fibrillation, stage IV decubitus ulcer	Yes	Yes; tracheostomy	Yes
3	75/F	Hypertension, diabetes, asthma, hyperthyroidism, congestive heart failure, dementia, anoxic encephalopathy, end stage renal disease (hemodialysis)	Yes	Yes; tracheostomy, PEG tube	Yes
4	54/F	Seizures, adrenal insufficiency, colon cancer	No	No	Yes
5	66/M	Hypertension, hyperlipidemia, chronic kidney disease, diabetes	Yes	No	Yes
6	79/M	Hypertension, coronary artery disease, end stage renal disease (hemodialysis), decubitus ulcer, stroke, breast cancer	Yes	Yes; PEG tube	Yes
7	72/M	Diabetes, hypertension, multiple myeloma (actively receiving chemotherapy)	No	No	Yes
8	78/M	Pneumococcal meningitis (1 mo earlier), respiratory failure status posttracheostomy and PEG placement, seizures, atrial fibrillation	Yes	Yes; tracheostomy, PEG tube	Yes
9	73/F	Hypertension, dementia, schizophrenia, stage IV decubitus ulcer	Yes	Yes; PEG tube	Yes
**Case-patient**	**Location at time of *C. auris* isolation**	**Concomitant positive cultures and antimicrobial drug therapy**	**Site of *C. auris* isolation/day of positive culture/day of first negative culture**	***C. auris* treatment course**	**Outcome**
Clinical course					
1	General medicine	Sputum: *Proteus mirabilis, Pseudomonas aeruginosa*; wound: ESBL *Escherichia coli*; pneumonia/infected wound: aztreonam, polymyxin B, vancomycin	Blood (+)/hospital day 1; blood (–)/hospital day 4	Intravenous micafungin, 100 mg/d x 28 d	Discharged to nursing home on hospital day 50

Case-patient	Age, y/sex	Medical history	Admitted from nursing home	Presence of invasive device	Recent broad-spectrum antimicrobial drug
2†	Intensive care unit	Sputum: *Acinetobacter calcoacticus, P. aeruginosa*; wound: *Klebsiella pneumoniae, P. aeruginosa*; pneumonia/infected decubitus ulcer: meropenem, gentamicin, polymyxin B, vancomycin	Blood (+)/hospital day 1; blood (–)/hospital day 2	Intravenous micafungin, 100 mg/d x 26 d	Died on hospital day 73
			Urine (+)/hospital day 39	Intravenous micafungin, 100 mg/d x 10 d plus intravenous liposomal amphotericin B, 4 mg/kg/d x 15 d	
			Blood (+)/hospital day 69; blood (+)/hospital day 70; blood (–)/hospital day 73	Intravenous liposomal amphotericin B, 4 mg/kg/d x 4 d (until patient died)	
3	General medicine	Sputum: *P. aeruginosa*, Stenotrophomonas. *maltophilia*; pneumonia: meropenem, vancomycin.ceftazidime, trimethoprim/sulfamethoxazole	Blood (+)/hospital day 27; blood (–)/hospital day 33	Intravenous micafungin, 100 mg/d x 12 d (as empiric treatment for suspected invasive candidiasis on hospital days 11–23)	Died on hospital day 38
				Intravenous micafungin, 100 mg/d x 9 d initiated after positive blood culture on hospital day 27 and continued until patient died	
4	Intensive care unit	Urine: *K. pneumoniae*; no concomitant antimicrobial drugs given	Blood (+)/hospital day 100; blood (–)/hospital day 103	Intravenous micafungin, 100 mg/d x 19 d	Discharged to nursing home on hospital day 161
5	Intensive care unit	Sputum: *A. baumannii*; pneumonia: meropenem , vancomycin, polymyxin B	Sputum/hospital day 30: positive for *C. albicans*;	Intravenous micafungin, 100 mg/d (as empiric treatment for suspected invasive candidiasis hospital days 30–44)	Discharged to nursing home for palliative care on hospital day 81
			Blood (+)/hospital day 45: positive for *C. auris*; blood (–)/hospital day 49	Intravenous micafungin dose increased to 150 mg/d in setting of positive blood culture for *C. auris* on hospital day 45; intravenous liposomal amphotericin B, 5 mg/kg/d added on to micafungin hospital day 60 because of persistent leukocytosis and febrile episodes; antifungal drugs discontinued after 30 d of micafungin and 19 d of liposomal amphotericin B	
6	General medicine	Two weeks before admission: blood: ESBL *K. pneumonia*; bacteremia: meropenem	Blood (+)/hospital day 1; blood (–)/hospital day 3	Intravenous micafungin, 100 mg/d x 14 d	Discharged to nursing home on hospital day 22
7	General medicine	Cultures: none; pneumonia: cefepime	Blood (+)/hospital day 1; blood (–)/hospital day 3	Intravenous micafungin, 150 mg/d x 14 d	Discharged home on hospital day 28
8	Nursing home (attached to hospital)	Sputum: ESBL *K. pneumoniae, P. aeruginosa*; blood: *A. baumanii*; pneumonia: cefepime, meropenem, vancomycin	Blood (+)/nursing home day 21; blood (–)/nursing home day 22	Intravenous micafungin, 150 mg/d x 18 d	Discharged to outside nursing home with palliative care on day 70

Case-patient	Age, y/sex	Medical history	Admitted from nursing home	Presence of invasive device	Recent broad-spectrum antimicrobial drug
9	Intensive care unit	Cultures: not available; septic shock: vancomycin and cefepime; vancomycin and meropenem	Blood(+)/hospital day 4; blood (–)/unavailable	Intravenous micafungin, 100 mg/d x 14 d	Discharged to nursing home with palliative care after completion of therapy. Hospital day of discharge was unavailable

*ESBL, extended-spectrum β-lactamase; IV, intravenous; PEG, percutaneous endoscopic gastrostomy.

†Case-patient 2 was initially given micafungin for 26 d for *C. auris* fungemia. On hospital day 36, the patient remained persistently febrile. Therefore, he was empirically given micafungin. On hospital day 39, his urine culture showed *C. auris* and amphotericin B was subsequently added to micafungin therapy. Combination therapy was continued for 7 days at which time micafungin was discontinued and the patient continued to receive amphotericin B for 8 additional days. On hospital days 69 and 70, blood cultures were again positive for *C. auris*. The patient was then given amphotericin B until he died.

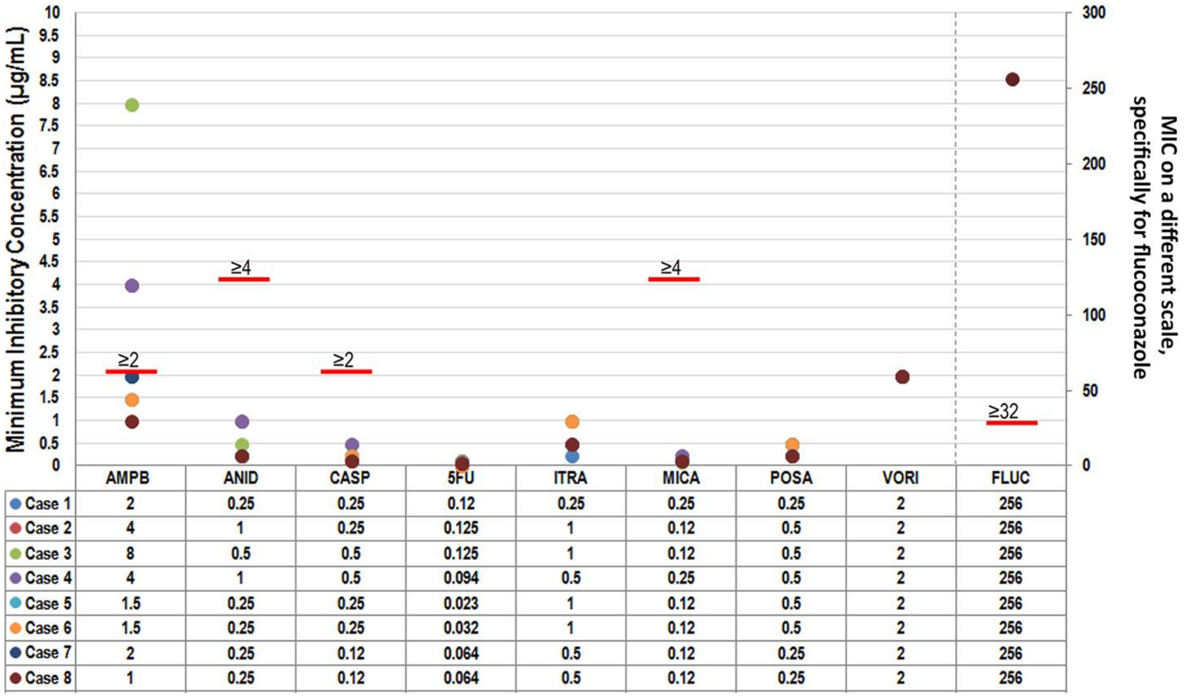

	AMPB	ANID	CASP	5FU	ITRA	MICA	POSA	VORI	FLUC
Case 1	2	0.25	0.25	0.12	0.25	0.25	0.25	2	256
Case 2	4	1	0.25	0.125	1	0.12	0.5	2	256
Case 3	8	0.5	0.5	0.125	1	0.12	0.5	2	256
Case 4	4	1	0.5	0.094	0.5	0.25	0.5	2	256
Case 5	1.5	0.25	0.25	0.023	1	0.12	0.5	2	256
Case 6	1.5	0.25	0.25	0.032	1	0.12	0.5	2	256
Case 7	2	0.25	0.12	0.064	0.5	0.12	0.25	2	256
Case 8	1	0.25	0.12	0.064	0.5	0.12	0.25	2	256

Appendix Figure. Antifungal susceptibility data for 8 case-patients during management of *Candida auris* fungemia at community Hospital, Brooklyn, New York, USA, 2016–2018. Red horizontal bars indicate MIC breakpoints per guidance of the Centers for Disease Control and Prevention (Atlanta, GA, USA). Tentative MIC breakpoints are not available for some antifungal agents included. AMPB, amphotericin B; ANID, anidulafungin; CASP, caspofungin; 5FU, 5-fluorouracil; ITRA, itraconazole; MICA, micafungin; POSA, posaconazole; VORI, voriconazole; FLUC, flucoconazole.
